# OncoCis: annotation of *cis-*regulatory mutations in cancer

**DOI:** 10.1186/s13059-014-0485-0

**Published:** 2014-10-09

**Authors:** Dilmi Perera, Diego Chacon, Julie AI Thoms, Rebecca C Poulos, Adam Shlien, Dominik Beck, Peter J Campbell, John E Pimanda, Jason WH Wong

**Affiliations:** Prince of Wales Clinical School and Lowy Cancer Research Centre, UNSW Australia, Sydney, 2052 Australia; Cancer Genome Project, Wellcome Trust Sanger Institute, Hinxton, CB10 1SA UK; Department of Haematology, Addenbrooke’s Hospital, Cambridge, CB2 0QQ UK; Department of Haematology, Prince of Wales Hospital, Sydney, 2031 Australia

## Abstract

Whole genome sequencing has enabled the identification of thousands of somatic mutations within non-coding genomic regions of individual cancer samples. However, identification of mutations that potentially alter gene regulation remains a major challenge. Here we present OncoCis, a new method that enables identification of potential *cis*-regulatory mutations using cell type-specific genome and epigenome-wide datasets along with matching gene expression *data.* We demonstrate that the use of cell type-specific information and gene expression can significantly reduce the number of candidate *cis*-regulatory mutations compared with existing tools designed for the annotation of *cis*-regulatory SNPs. The OncoCis webserver is freely accessible at https://powcs.med.unsw.edu.au/OncoCis/.

**Electronic supplementary material:**

The online version of this article (doi:10.1186/s13059-014-0485-0) contains supplementary material, which is available to authorized users.

## Background

Research into cancer-causing mutations has focused primarily on protein-coding mutations owing to difficulties associated with identifying and interpreting causality of non-coding mutations. However, projects such as ENCODE [1] and the Human Epigenome Atlas [2] have led to the generation of genome-wide datasets that have contributed to our understanding of the non-coding regions of the human genome. The integration of these datasets shed light on the functions of non-coding sequences, gene regulatory modules and epistatic interactions underlying disease associations. Moreover, the rapid advancement of sequencing technologies and the rapid drop in sequencing costs have now made it feasible to sequence whole genomes of large numbers of cancer samples. Nevertheless, even though cancer genomes are being sequenced at an accelerated pace, annotation of mutations and inference of their functional significance remain challenging. Whilst a myriad of tools are now available for the annotation of protein-coding mutations (for example, [3–6]), identification of *cis-*regulatory mutations remains a major challenge.

In recent years, computational approaches have been developed to help identify non-coding germline sequence variants that have the potential to modify gene regulation. HaploReg [7] was one of the first databases available for annotating variants in non-coding regions of the genome. Using linkage disequilibrium information from the 1000 Genomes Project, it allows for the visualization of linked SNPs and small indels along with their predicted chromatin state, their sequence conservation across mammals, and their effect on regulatory motifs. It included a library of SNPs (based on dbSNP 137), motif instances (based on position-weighted matrices discovered from ENCODE experiments), enhancer annotations (adding 90 cell types from the Roadmap Epigenome Mapping Consortium), and expression quantitative loci (eQTLs from the GTex eQTL browser [8]). rSNPBase [9] is a similar database which curates and annotates regulatory SNPs. It uses data from genome-wide experiments from the ENCODE project to predict regulatory elements which are then used to annotate the rSNPs. These rSNPs are mapped to the gene that it may regulate by considering various regulation mechanisms like proximal/distal regulation and post-transcriptional regulation. It also takes into account the linkage disequilibrium correlations between SNPs in order to associate the regulatory element with a SNP-set as opposed to a single SNP. Spatio-temporal and experimental eQTL labels are also provided in rSNPBase annotations. The main limitation of both the above databases is that they are only suitable for SNPs that have already been identified and catalogued. As such, they are not suitable for the study of novel somatic mutations in non-coding regions.

More recently, a number of tools have been developed that can be used to interrogate the *cis-*regulatory potential of novel non-coding variants in the human genome. These include RegulomeDB [10], Funseq [11] and GAWVA [12], all of which leverage a large number of ENCODE datasets to infer the potential impact of a variant on the *cis-*regulation of a gene. RegulomeDB provides a heuristic scoring system which classifies the regulatory potential of predicted regions in the genome. SNPs that fall within these regions will be associated with this score as an indication of its likelihood of affecting gene regulation. GAWVA uses a similar approach, but implements a computational model trained using known non-coding disease SNPs to provide a classifier score which can be used to indicate the *cis-*regulatory potential of novel SNPs and mutations. Finally, Funseq leverages an observation that genomic regions with a high concentration of rare SNPs indicate a higher degree of negative selection and are thus more likely to be functionally important such that the function of the region is more ‘sensitive’ to sequence variation [11]. Therefore, in addition to using ENCODE datasets, Funseq determines whether a particular variant falls within ‘sensitive’ regulatory regions in the human genome.

While all the above-mentioned methods can be used for the annotation of somatic mutations, they are better suited for the annotation of germline variants as they lack the ability to assess mutations in a tissue/cell-specific context. Unlike SNPs, which have the potential to exert a phenotype across all cell types, somatic mutations arising in cancer are confined to altering gene expression within cancer cells that harbor the mutation. As such, existing tools targeted at annotation of SNPs are of limited value to the end user seeking to prioritize the impact of a mutation on the aberrant expression of a gene in a particular tumor. Furthermore, other important features for assessing the potential regulatory impact of mutations, including transcription factor binding motif creation and the integration of matched gene expression data, are not available in existing tools.

To this end, we have developed OncoCis, a user-friendly webserver for researchers, to annotate *cis-*regulatory cancer mutations in a tissue/cell-specific manner. Importantly, a set of stringent annotation methods, including location of flanking histone marks, motif matching and integration of gene expression, have been developed to increase the accuracy of the mutation annotations. To validate OncoCis, we first demonstrated its ability to correctly annotate the well-studied *TERT* promoter mutations [13]. We then compared OncoCis with RegulomeDB and Funseq in their ability to annotate non-coding mutations derived from whole genome sequencing data from 17 breast cancer samples [14]. Finally, using a specific example from the breast cancer dataset, we highlight the ability to use OncoCis to identify potential *cis-*regulatory mutations for further analysis.

## Results and discussion

### Overview of OncoCis

**Figure 1:**
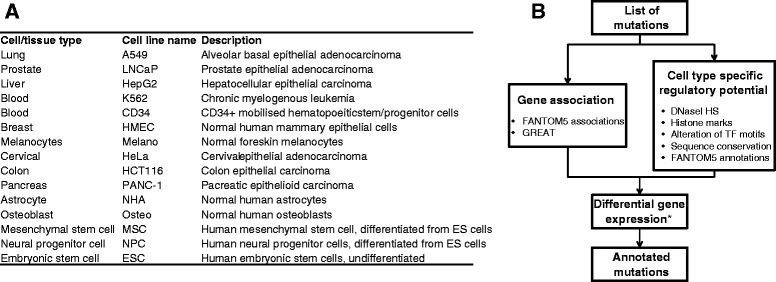
**Overview of OncoCis datasets and algorithms. (A)** Summary of representative cell/tissue types available for annotation of mutations. **(B)** Schematic diagram illustrating the mutation annotation process implemented in OncoCis. *Differential gene expression is only performed if gene expression data are available. ES, embryonic stem; HS, hypersensitive; TF, transcription factor.

OncoCis integrates publicly available datasets representing a wide range of cancer types from genome-wide chromatin accessibility and histone modification profiles obtained from ENCODE [1] and the Human Epigenome Atlas [2] to identify mutations that occur within potential *cis-*regulatory regions (see Figure 1A for a list of cell types). These mutations are further annotated with sequence conservation scores and searched for possible elimination or creation of transcription factor consensus binding motifs from the JASPAR 2014 database [15]. Enhancer-transcription start site (TSS) associations generated by the FANTOM5 consortium [16] and the GREAT tool [17] are used to map mutations to the most likely gene on which it may have a regulatory impact. Finally, if gene expression data are available, differential expression will be calculated between samples with and without potential *cis-*regulatory mutations for each gene linked with a particular mutation (Figure 1B).

**Figure 2:**
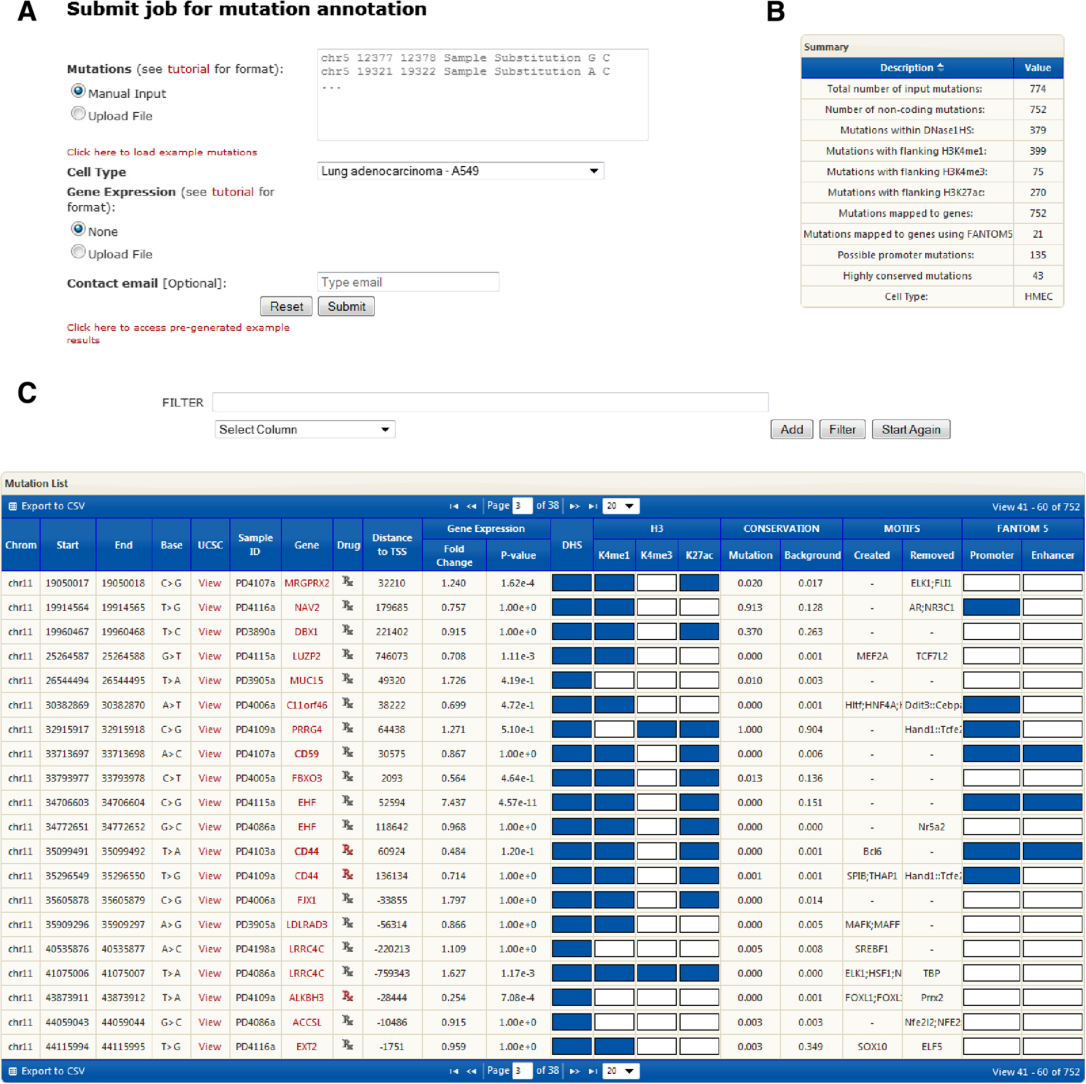
**Screenshot of the OncoCis webserver interface. (A) **Input fields. **(B)** Summary output. **(C)** Annotated mutations.

To facilitate the use of OncoCis, a user-friendly interface is provided to enable a user to upload a list of candidate mutations, select a specific tissue/cell type representative of the cancer type from which the mutations are derived and upload associated gene expression data if available (Figure 2A). Following the analysis of the mutations by OncoCis, a summary of the annotations is provided (Figure 2B). The resulting individual mutation annotations are displayed in an interactive and filterable table (Figure 2C). The table provides a hyperlink to visualize mutations and associated contextual epigenomic profiles within the UCSC genome browser. Furthermore, to enhance utility of annotations associated with mutations, OncoCis provides a hyperlink to DGIdb [18] and directly indicates whether an associated gene is potentially drugable. This is particularly useful for researchers to prioritize genes with therapeutic potential. Finally, the set of mutation annotations can be conveniently exported as text files for further offline analysis.

### Validation of OncoCis using *TERT* promoter mutations

To validate the ability of OncoCis to identify candidate *cis-*regulatory mutations, OncoCis was used to annotate the *TERT* promoter mutations, which are currently the best established example of recurrent *cis-*regulatory mutations found across a variety of cancers and in particular cancers of the central nervous system [13,19–21]. OncoCis annotated the two *TERT* promoter mutations (chr5:1,295,228 G > A and 1,295,250 G > A) as being -66 bp and -88 bp from the *TERT* TSS, respectively. The mutations fall within a DNase I hypersensitive site (DHS) and flank a H3K4me3 histone mark of neural progenitor cells. Furthermore, the mutated bases created an ETS factor binding site in both cases, which was consistent with previous studies of these mutations [13,19–21] (see Table 1 for full OncoCis annotation). The same mutations were also analyzed using RegulomeDB and Funseq (Table 1). RegulomeDB found the mutations to be in categories 2b and 4, meaning that it only identified one of the sites as likely to affect transcription factor binding. In terms of Funseq annotations, neither of the mutations was within a ‘sensitive’ region. This suggests that while ‘sensitive’ regions may indicate functionally important genomic regions, causal *cis-*regulatory mutation can occur outside these regions. Significantly, since both RegulomeDB and Funseq only evaluate the removal of transcription factor binding motifs, neither tool was able to suggest the creation of an ETS binding site by the *TERT* promoter mutations.

**Table 1:** **OncoCis annotation of the** ***TERT*** **promoter mutations chr5:1,295,228 G > A and 1,295,250 G > A**

									**Motifs**		**Fantom5**		**RegulomeDB**	**Funseq**
**Chromosome**	**Position**	**Gene**	**Distance to TSS**	**DHS**	**H3K4me1**	**H3K4me3**	**H3K27ac**	**Conservation mutated base**	**Created**	**Removed**	**Promoter**	**Enhancer**	**Category**	**Sensitive?**
Chr5	1,295,228	*TERT*	-66	1	0	1	0	0.008	*ELK1;ELF1;FLI1;ELK4;GABPA*	*TFAP2A*	1	0	2b	No
Chr5	1,295,250	*TERT*	-88	1	0	1	0	0	*ELK1;ELF1;FLI1;ELK4;GABPA*	*-*	1	0	4	No

The category and whether the mutation falls within a ‘sensitive’ region as defined by RegulomeDB and Funseq, respectively, are also shown.

### Cell type-specific information reduces candidate *cis-*regulatory mutations

**Figure 3:**
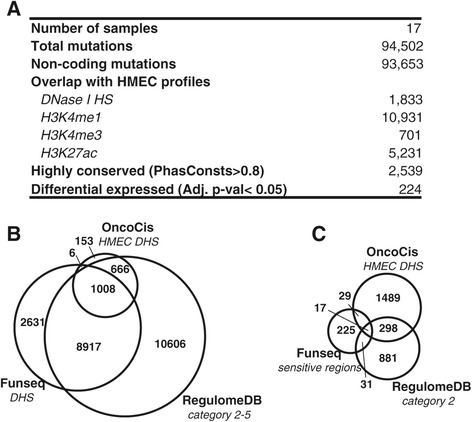
**Analysis of non-coding mutations using OncoCis, RegulomeDB and Funseq. (A)** A summary of mutation annotations from 17 whole breast cancer genomes by OncoCis. **(B)** Overlap of mutations annotated as within DHSs from the whole breast cancer genomes using OncoCis (from HMEC line), RegulomeDB (mutations within categories 2 to 5) and Funseq (mutations within DHSs). **(C)** Overlap of mutations annotated as likely to be functionally important in RegulomeDB (mutations within category 2), Funseq (mutations within ‘sensitive’ regions) and mutations within cell-specific (HMEC) DHS regions by OncoCis.

To further validate the ability of OncoCis to correctly annotate mutations and to demonstrate the value of using cell type-specific *cis-*regulatory information, we analyzed mutations from 17 whole-genome sequenced breast cancer samples [14]. In total there were 94,502 mutations across all samples, of which 93,653 were within non-coding regions of the genome (Figure 3A). To assess the cell type-specific epigenome profiles most relevant to breast cancer, data from the human mammary epithelial cell (HMEC) line was used. Of all mutations, 1,833 fell within a HMEC DHS (Figure 3A). Comparison of the same set of mutations that fell within DHS across all available cell types as determined by RegulomeDB and Funseq showed that, as expected, many more mutations were annotated within a DHS (Figure 3B; see Additional file 1 for full analysis output from OncoCis, RegulomeDB and Funseq). Most mutations (1,680, 91.7%) determined by OncoCis to fall within HMEC DHSs were present in the non-cell type-specific sets identified by RegulomeDB and Funseq. This overlap was significantly greater than randomly drawn mutations from the dataset (mean 1,063, 57.0%, *P* <0.001, one-sample *t*-test), demonstrating that the DHS annotations from OncoCis are consistent with non-cell type-specific DHS annotations. Mutations falling within HMEC DHSs unique to OncoCis are likely due to the fact that this dataset was from the Human Epigenome Atlas, which is not part of the ENCODE data used by RegulomeDB or Funseq. Importantly, when only high-priority mutations were selected for RegulomeDB (category 2; note that there are no mutations in category 1 as the input data contain only somatic mutations and are therefore not expected to be linked to eQTLs) or Funseq (variants within ‘sensitive’ regions under strong evolutionary selection), only a small portion (25.7% and 15.2% for RegulomeDB and Funseq, respectively) of these mutations fell within HMEC DHSs as determined by OncoCis (Figure 3C). This illustrates that by incorporating cell type-specific information, OncoCis was able to eliminate a considerable number of mutations that would otherwise have been prioritized by using either RegulomeDB or Funseq.

### Incorporation of matched gene expression data helps prioritize *cis-*regulatory mutations

As matching gene expression data were available for the 17 breast cancer samples, OncoCis was able to calculate expression differences of genes associated with mutations in a DHS, between samples with and without a particular mutation. In total, 18 mutations were found to potentially alter *cis-*regulation as they were associated with the following features; (a) there was altered expression (adjusted *P*-value <0.05) of the associated gene in the sample with the mutation compared with samples without the mutation, (b) the mutation resided in a region with at least one active histone mark, (c) mammalian sequence conservation of >0.8 and, (d) caused the gain or loss of at least one transcription factor binding motif (Table 2). Of the 18 mutations, all were annotated as category 5 or above by RegulomeDB (Table 2). By linking these mutations to a change in gene expression, RegulomeDB would have effectively categorized all of these as ‘likely to affect binding and linked to expression of a gene target’ (category 1) [10]. This again illustrates the consistency of OncoCis annotations with RegulomeDB annotations. Conversely, however, if RegulomeDB alone was used, a total of 1,227 category 2 mutations would have been identified (Figure 3C), of which only 6 were amongst the 18 OncoCis prioritized mutations. Similarly, of 302 mutations determined to be within a ‘sensitive’ region by Funseq, only 3 were within the OncoCis prioritized mutations. While ‘sensitive’ regions under selective pressure are more likely to be important [11], not all *cis-*regulatory mutations necessarily fall within one of these regions as shown in the *TERT* promoter mutations earlier. More generally, a similar pattern was found when comparing any mutations annotated by OncoCis as being associated with differential expression against annotations from RegulomeDB and Funseq (Additional file 2). Taken together, this demonstrates that, using a more stringent annotation methodology, OncoCis has significant advantages in identifying relevant mutations with high *cis-*regulatory potential.

**Table 2:** **OncoCis annotations of mutations from 17 whole breast cancer genomes sorted by differential gene expression** ***P*** **-value of the sample where there is an associated mutation and the samples without any associated mutation for a particular gene**

				**Distance**	**Gene expression**						**Conservation**	**Motifs**		**Fantom5**		**RegulomeDB**	**Funseq**
**Chromosome**	**Position**	**Sample ID**	**Gene**	**to TSS**	**Fold change**	***P*** **-value**	**DHS**	**H3K4me1**	**H3K4me3**	**H3K27ac**	**Mutated base**	**Created**	**Removed**	**Promoter**	**Enhancer**	**Category**	**Sensitive?**
Chr6	71,108,774	PD4006a	*COL9A1*	-95,988	100.12	1.39E-23	1	1	1	1	1	*Hand1::Tcfe2a*	*Klf1*	1	0	4	No
Chr1	160,094,923	PD4116a	*ATP1A2*	-9,404	71.15	2.99E-19	1	1	0	1	0.981	*ELF1;Hltf*	*SP1;ZEB1*	1	0	4	No
Chr7	92,347,495	PD4107a	*CDK6*	118,446	5.06	7.75E-07	1	1	1	1	1	-	*NFIC;THAP1*	1	0	4	No
Chr9	109,651,512	PD4006a	*ZNF462*	-26,135	4.79	2.27E-06	1	1	0	1	0.993	-	*EHF;Erg;FLI1;PPARG::RXRA;.*	0	0	5	No
Chr1	208,412,585	PD4116a	*PLXNA2*	5,080	1.63	4.17E-06	1	1	0	1	0.993	*Nobox;Hltf*	-	0	1	4	No
Chr4	7,5560,994	PD4103a	*BTC*	158,888	2.38	7.39E-05	1	1	0	0	0.938	-	*CREB1;Mafb*	0	0	4	No
Chr5	97,643,723	PD4109a	*RGMB*	461,275	2.50	2.47E-04	1	1	0	1	0.801	-	*ARID3A*	0	1	3a	No
Chr17	2,080,270	PD4005a	*HIC1*	-120,667	1.29	4.02E-04	1	1	0	1	1	*NFIC*	-	0	0	5	No
Chr16	57,334,425	PD4115a	*PLLP*	-15,841	2.09	8.51E-04	1	1	1	1	1	-	*TFAP2C*	1	0	4	Yes
Chr2	219,147,431	PD4198a	*TMBIM1*	9,849	1.65	2.23E-03	1	1	0	1	0.973	*Foxd3*	*NFATC2;Erg*	1	0	5	No
Chr8	100,811,550	PD4116a	*COX6C*	94,692	2.46	2.63E-03	1	1	0	0	1	-	*NFKB1;Stat4;Spi1;Bcl6*	0	0	2b	No
Chr14	37,612,228	PD4115a	*SLC25A21*	29,637	1.54	3.15E-03	1	1	0	0	1	*Hand1::Tcfe2a*	*RUNX1;RUNX2;FOXI1*	0	0	2b	No
Chr1	185,688,035	PD4005a	*HMCN1*	15,647	0.61	4.98E-03	1	1	0	1	0.997	*FOXP1;FOXL1*	-	0	0	5	No
Chr2	208,890,286	PD3904a	*PLEKHM3*	-2	1.48	1.09E-02	1	1	1	1	0.998	-	*ELF5;GABPA;FLI1;ELK4;ELK1*	1	0	2b	No
Chr10	93,058,182	PD4005a	*PCGF5*	-77,814	0.65	2.28E-02	1	1	0	0	0.935	-	*Zfx*	1	0	2a	No
Chr7	22,617,382	PD4107a	*IL6*	-149,383	2.29	2.62E-02	1	1	0	1	1	*Hltf;CEBPA;CEBPB*	*AR*	1	1	2a	Yes
Chr6	26,533,145	PD4192a	*HMGN4*	5,426	0.49	2.62E-02	1	1	0	0	0.979	-	*Zfx*	0	1	2b	Yes
Chr22	31,644,327	PD4103a	*LIMK2*	-36,078	0.79	3.43E-02	1	1	1	1	0.989	-	*Klf4;SP2;SP1;KLF5;Klf1;ELK1*	1	0	3a	No

All mutations in the list are selected based on having a DHS, either a H3K4me1 or H3K4me3 histone mark, conservation (phastCons) >0.8 and the creation or removal of at least one transcription factor binding motif. The category and whether the mutation falls within a ‘sensitive’ region as defined by RegulomeDB and Funseq, respectively, are also shown.

### OncoCis identifies a *cis-*regulatory mutation that potentially perturbs the expression of *CDK6*

**Figure 4:**
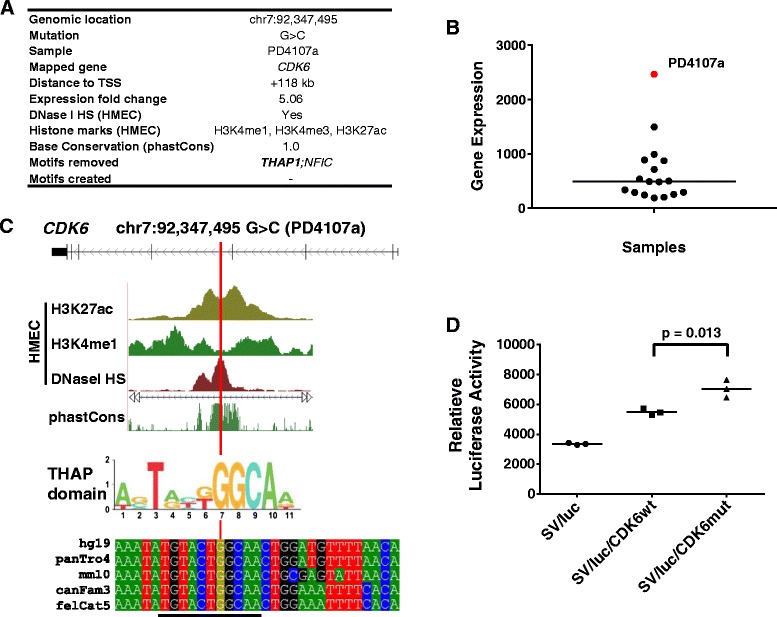
**Example of a potential** ***cis-*** **regulatory mutation associated with** ***CDK6*** **. (A)** Summary of OncoCis annotations of a mutation that is strongly suggestive of altering ***cis-***regulation of *CDK6*. **(B)** Distribution of expression level of *CDK6* in the sample with the potential *cis-*regulatory mutation (red) and samples without *cis-*regulatory mutations of *CDK6* (black). **(C)** Illustration of the location of the potential *cis-*regulatory mutation within intron 4 of *CDK6* along with its relative position to HMEC DHS, H3K4me1 and H3K27ac peaks. The potential for the mutation to alter the THAP transcription factor consensus binding site is shown along with cross-species conservation of the mutated base and its adjacent sequences in mammals. **(D)** Luciferase reporter assays for the putative CDK6 enhancer (chr7:92,347,263-92,347,759) showing control (SV/luc), wild-type sequence (SV/luc/CDK6wt), and chr7:92,347,495 G > C mutation (SV/luc/CDK6mut) in the HCC1143 breast cancer cell line. The results depicted are representative of three independent experiments.

To illustrate how OncoCis might be used to select potential *cis-*regulatory mutations for further experimental analysis, of the 18 prioritized mutations, the G > C substitution at chr7:92,347,495 in one of the samples (PD4107a) was examined. OncoCis determined this mutation to be associated with a five-fold up-regulation of *CDK6* (Figure 4A,B) when compared with the samples without the mutation. *CDK6* is a gene that activates cell proliferation [22] and is commonly found to be up-regulated in cancers, including breast cancer [23]. Examination of the location of the mutation showed that it fell within a highly conserved region in intron 4 of *CDK6* within a HMEC DHS flanked by H3K4me1 and H3K27ac. These features suggest that the mutation was located within a potential regulatory region of *CDK6* (Figure 4C). Furthermore, the substitution of G > C was predicted to disrupt the consensus binding motif for the transcription factor THAP. The THAP family of transcription factors consists of 11 factors that have been shown to play a variety of roles in controlling cell proliferation, cell cycle progression, angiogenesis, apoptosis and epigenetic gene silencing [24]. Examination of the set of *THAP* factor expression across the breast cancer samples showed that they were ubiquitously expressed across the samples (Additional file 3). Importantly, there is strong evidence in the literature that THAP1, 5, 7 and 11 act as negative regulators [24–27] which is consistent with the loss of THAP binding caused by the G > C substitution resulting in increased *CDK6* expression.

To further validate that the mutation alters *CDK6* regulation, an enhancer luciferase reporter assay was performed to compare the activity of the wild-type and mutant sequences. While both the wild-type and the mutant sequence enhanced the control SV promoter activity, the mutant further significantly increased the relative luciferase signal by 1.28-fold over the wild-type (*P* =0.013, unpaired *t*-test; Figure 4D). This is consistent with the increased expression seen in this sample relative to the other breast cancer samples. Since FANTOM5 data suggest that certain cell lines may have alternative *CDK6* transcripts arising from this regulatory region, we analyzed RNA-seq data from this breast cancer sample (PD4107a) but found no evidence of alternative transcripts initiating from this intronic enhancer (Additional file 4).

Four of the 18 candidate mutations were associated with known cancer driver genes: *CDK6*, *IL6*, *COX6C* and *HIC1*. The predicted alterations in *cis*-regulation as a consequence of these mutations were consistent with the known function and altered expression of *IL6* and *COX6C* but not *HIC1* (Additional file 5), highlighting the need for validation in a relevant experimental system. Taken together, the examples demonstrate the utility of OncoCis for prioritizing potential *cis*-regulatory mutations in cancer for further analysis and validation.

## Conclusions

As whole cancer genome sequencing becomes increasingly commonplace, there is an urgent need to enable the prioritization of functionally relevant mutations within non-coding regions of the genome for functional validation. Importantly, the application of OncoCis is not limited to somatic mutations and can also be used for the annotation of SNPs especially where cell type-specific gene regulation may be relevant. As more regulatory variants and mutations are discovered, there will be further potential to develop models for scoring and prioritizing *cis*-regulatory mutations. Taken together, OncoCis provides an avenue to uncover *cis*-regulatory mutations and it is hoped that in the process it will help discover new non-coding cancer driver mutations.

## Materials and methods

### Data sources

Chromatin accessibility data (DNase-seq) and histone modification (ChIP-seq) data for each of the cell lines were obtained from ENCODE [1] or the Human Epigenome Atlas [2]. A summary of datasets and associated accessions used by OncoCis are listed in Additional file 6. For datasets downloaded from ENCODE, broadpeaks of DNase-seq and histone ChIP-seq data are directly used for annotation. For all other datasets, raw sequences files in SRA format were obtained and converted to fastq format using fastq-dump (version 2.3.2). Fastq files were aligned using BWA (version 0.7.5) [28] using default parameters. For peak calling, the findPeaks tool within the HOMER suite [29] was used with the ‘style’ option set to ‘histone’.

### DNase I hypersensitivity sites: DNase-seq

OncoCis uses DNase-seq data to determine DHSs in order to identify user defined mutations that fall within chromatin accessible regions of the genome. DHS peak lists are defined as described above. OncoCis uses bedtools intersect [30] to determine whether or not a mutation falls within a DHS.

### Histone modifications: ChIP-seq

OncoCis annotates whether user-defined mutations are adjacent to specific histone marks, specifically H3K4me1, H3K4me3 and H3K27ac, which are generally associated with enchancers, promoters and active *cis-*regulatory regions, respectively [31]. In order to determine the optimal region for search for histone marks flanking mutations, the distribution of histone ChIP-seq signals adjacent to DHSs was analyzed. The analysis revealed that the optimum region for searching for flanking histone marks is 150 to 500 bp on either side of the center of a DHS (Additional file 7). As a result, mutations falling within ±150 to 500 bp of a histone ChIP-seq peak are annotated as flanking the respective histone mark. For mutations that fall within a DHS, the center of the DHS peak is used to define the ±150 to 500 bp region for seeking the presence of a histone peak, otherwise, the ±150 to 500 bp region is defined from the location of the mutation.

### Transcription factor motif search

For each mutation, both the wild-type and mutant DNA sequence are generated over a region of ±20 bp of the mutation site. Each sequence is individually searched for known consensus transcription factor binding motifs using the Possum tool [32] against the JASPAR 2014 database [15]. To increase the accuracy of the motif search, only mammalian transcription factors represented by more than 20 sequences from the JASPAR database are used (171 in total). Possum predicts transcription factor binding sites in a DNA sequence using position-specific scoring matrices and calculates a log-likelihood ratio score for each transcription factor binding sequence matrix model against the DNA sequence such that:

$$ Score= \log {}_2\kern0.5em \left({\displaystyle {\prod}_{k\kern0.5em =\kern0.5em 1}^W}\frac{q\left(k,{L}_k\right)}{p\left({L}_k\right)}\right) $$

where *W* is the size of the motif, *q*(*k*,*L*_*k*_) is the probability of the nucleotide at *L*_*k*_ at position *k* of the matrix and *p*(*L*_*k*_) is the background probability of base *L*_*k*_. In OncoCis, a score cutoff of >5 is implemented (default of Possum), *L*_*k*_ is set uniformly to ¼ and no pseudocounts are added to model matrices.

In order to increase the stringency of potential motif matches and to help users select mutations that are most likely to affect gene regulation, an additional filter was used to select DNA sequences where all well conserved motif positions (defined as those where a particular base is present at >80% frequency) match perfectly to the query DNA sequence. No significant correlation was found between the numbers of motif matches and the number of well conserved positions within a motif (Additional file 8). The use of variation at well conserved motif bases as a criterion for identifying variants that affect *cis-*regulation was used recently to discover a SNP that altered binding of TP53, which is linked to increased cancer risk [33].

Finally, the candidate transcription factors binding the wild-type and mutant sequences are compared such that wild-type-specific transcription factor binding motifs are reported as motifs removed and mutant specific transcription factor binding motifs are reported as motifs created.

### Mapping mutations to genes

The mutations are mapped to the gene which it is most likely be affecting using a combination of FANTOM5 enhancer-TSS interaction data [16] and predictions from the GREAT tool [17], which associates genomic locations with genes based on proximity, but also incorporates experimental 3C chromosome conformation capture information where available. If a mutation falls within a FANTOM5-predicted enhancer which has an association with the TSS of a gene, the mutation will be mapped to that gene. Otherwise, the mutation will be mapped to the closest gene within 1 Mbp using GREAT.

### Conservation of mutated base(s)

For all mutations, the phastCons score [34] from mammals is used for determining the conservation of the mutated base in the case of substitutions and bases in the case of deletions. For deletions, the reported conservation value is the average of the phastCons score across all deleted bases. For insertions, no conservation value is reported. A background mutation value is also calculated by OncoCis using the average conservation value of all bases within ±20 bp of the mutation site.

### Gene expression analysis

If gene expression data have been provided by the user, fold change and a *P*-value associated with that fold change will be calculated for each gene that has been associated with a mutation. For the computation of differential gene expression, only mutations that have been associated with a gene but also falling within a DHS are evaluated. For a particular gene, all samples with a mutation within one DHS will be categorized as mutant samples while the samples without any mutations within that DHS are categorized as non-mutant. The gene expression level of each mutant will be compared with the median of all non-mutant samples to calculate fold change. A two-sided *t*-test is conducted between the expression level of each mutant sample and non-mutant samples to obtain a *P*-value for the change in gene expression. A Bonferroni correction using the number of DHS-associated mutations is finally applied to obtain a false discovery rate-adjusted *P*-value. If the user has gene expression data of additional normal (non-cancer) samples, these can be also provided and will be automatically categorized as additional non-mutant samples as they will not be associated with any mutations.

### FANTOM5 enhancer/promoter transcription start site predictions

The mutations are further annotated with FANTOM5 enhancer [16] and promoter TSS predictions [35] to provide additional evidence for a mutation to affect gene *cis-*regulation. For OncoCis, global permissive promoter and enhancer datasets were used because CAGE TSSs from individual cell lines/tissues only arise from active *cis-*regulatory elements and cannot be used to identify promoters and enhancers that are poised or repressed [35]. Specifically, the enhancer dataset was obtained from [36] and the promoter TSS dataset from [37]. All enhancer transcript and promoter TSS predictions were extended by 500 bp in both directions, since FANTOM5 annotations provide the location of the transcript rather than the actual enhancer/promoter. Bedtools intersect [30] is used determine the overlap.

FANTOM5 enhancer and promoters have been shown to be complementary to ENCODE regulatory datasets, but is generally more stringent [35]. Across the breast cancer mutation dataset, 238 out of 301 (79.1%) mutations within a DHS and flanking a H3K4me3 mark overlap with a FANTOM5 promoter, while 582 out of 1,490 (39.1%) mutations within a DHS and flanking a H3K4me1 mark overlap with a FANTOM5 enhancer. This is broadly in line with the finding that 30 to 40% of ENCODE TSS segments overlap with FANTOM5 data [35].

### Analysis of breast cancer mutations using OncoCis and comparison with RegulomeDB and Funseq

To assess the function and annotations of OncoCis in comparison with RegulomeDB [10] and Funseq [11], mutations from 17 whole breast cancer genome sequenced samples [14] with matching gene expression data were used. RegulomeDB and Funseq were chosen as they are currently the only two tools which provide a webserver for the annotation of novel mutations. For OncoCis, the mutation list and gene expression datasets were uploaded to the webserver. HMECs were selected as the cell type. For RegulomeDB, the list of mutations was submitted to the webserver in bed format (there are no further parameters required) and the full output of the results was exported. For Funseq the list of mutations was also submitted to the webserver in bed format with the option for minor allele frequency set to 0. The mutations annotations from the three tools were compiled for further analysis and comparison (Additional file 1).

To evaluate the significance of the overlap between mutations within HMEC DHS regions from OncoCis in comparison with mutations within DHS annotations from RegulomeDB and Funseq, a bootstrapping analysis was performed. To achieve this, 1,833 mutations (the total number of mutations that were found to overlap with HMEC DHSs by OncoCis) were randomly selected from the full set of 93,459 non-coding mutations and the overlap with the combined DHS annotations from RegulomeDB and Funseq was assessed. This procedure was repeated 1,000 times to give an average overlap of 1,063 mutations (standard deviation of 21) with RegulomeDB or Funseq, which is significantly less than the overlap of 1,680 mutations (*P* <0.001, one sample *t*-test) also with DHS annotation by RegulomeDB or Funseq as shown in Figure 3B.

### Luciferase report assay

The minus strand of a conserved DHS (chr7:92,347,263-92,347,759) flanking the chr7:92,347,495 G > C mutation was synthesized as both wild-type (SV/luc/CDK6wt) and mutant (SV/luc/CDK6mut) versions using GeneArt Strings (Life Technologies, Mulgrave, VIC, Australia) and cloned into the downstream multiple cloning site of pGL2P (Promega, Alexandria, NSW, Australia). HCC1143 breast cancer cells were cultured in RPMI1640 media supplemented with 15% fetal bovine serum, HEPES, sodium pyruvate, glutamine, and penicillin/streptomycin. For transfections, cells were seeded at 4 × 10^5^/well cells in six-well plates and transfected the following day using Lipofectamine2000 (Life Technologies) with 2 μg of *CDK6* reporter construct or vector control (SV/luc) and 0.5 μg of pEFBOS LacZ. At least two separate experiments were performed in triplicate wells. After 48 hours, cells were lysed and luciferase and β-galactosidase activity was assayed as described [38]. To control for transfection efficiency, relative luciferase activity was calculated as the ratio of luciferase to LacZ activity.

### Webserver implementation

OncoCis is implemented as a webserver at [39]. The backend application has been implemented using Java (1.6) with datasets stored in a MySQL database. PHP was used to implement the front-end web interface. The OncoCis standalone application with the source code is available at the server website.

## Additional files

Additional file 1:
**Annotations of all non-coding mutations in the 17 breast cancer samples using OncoCis, RegulomeDB and Funseq.**
Additional file 2:
**Comparison of differential gene expression and transcription factor binding site creation annotations in OncoCis and annotations from RegulomeDB and Funseq.**
Additional file 3:
**Expression of THAP transcription factors across the breast cancer samples.**
Additional file 4:
**Analysis of RNA-seq data to identify potential alternative TSS in** ***CDK6.***
Additional file 5:
**Examples of other candidate mutations from the breast cancer dataset prioritised by OncoCis.**
Additional file 6:
**Accession of datasets implement in OncoCis.**
Additional file 7:
**Illustrating the distribution of histone profiles adjacent to DHSs.**
Additional file 8:
**Analysis of the effect of number of well conserved positions in motifs against its frequency of being found to be created/removed.**

## Abbreviations

bp: base pair
DHS: DNase I hypersensitive site
eQTL: expression quantitative locus
HMEC: human mammary epithelial cell
SNP: single-nucleotide polymorphism
TSS: transcription start site
