# Supplementary material for: OncoCis: annotation of cis-regulatory mutations in cancer
Source: Genome Biol. 2014 Oct 9;15(10):485. doi: 10.1186/s13059-014-0485-0 (PMC4224696; doi:10.1186/s13059-014-0485-0)
Supplement: Additional file 2: — Comparison of differential gene expression and transcription factor binding site creation annotations in OncoCis and annotations from RegulomeDB and Funseq. [file 13059_2014_485_MOESM2_ESM.docx]

**Additional File 2. Comparison of differential gene expression and transcription factor binding site creation annotations in OncoCis and annotations from RegulomeDB and Funseq.**

To evaluate generally the distribution of novel annotation features from OncoCis including differential gene expression and transcription factor binding site creation, a comparison was performed between these annotations and annotations from RegulomeDB and Funseq. In total OncoCis annotated 224 mutations with differential expression (by default all mutations evaluated for association with differential expression must also fall within a DHS) and 857 mutations with transcription factor binding site creation + falling within a DHS (Additional File 2 Figure).


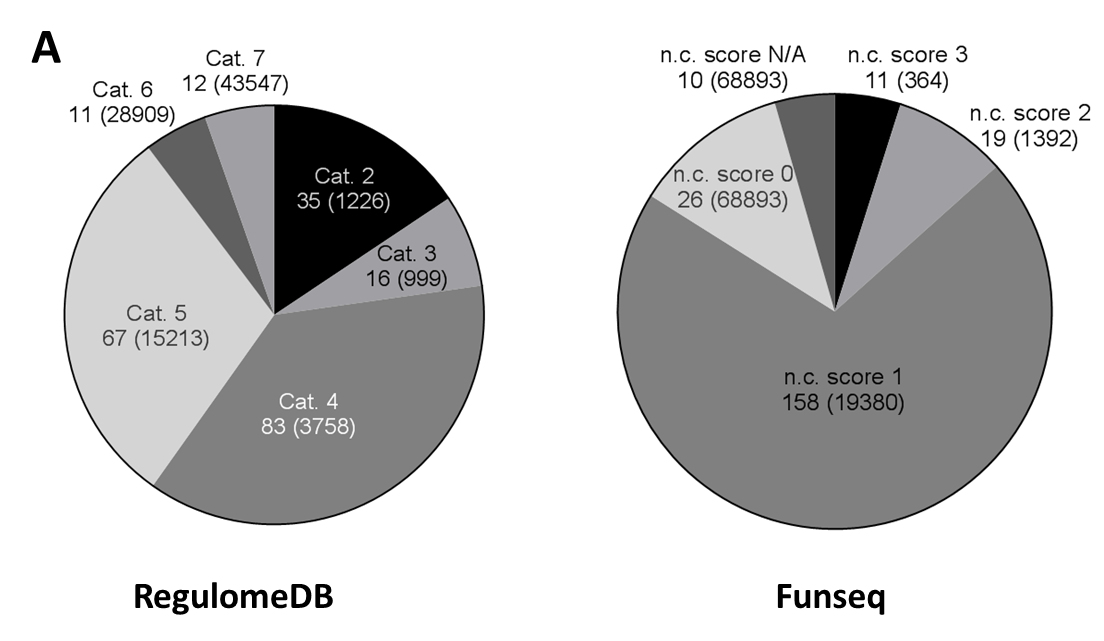

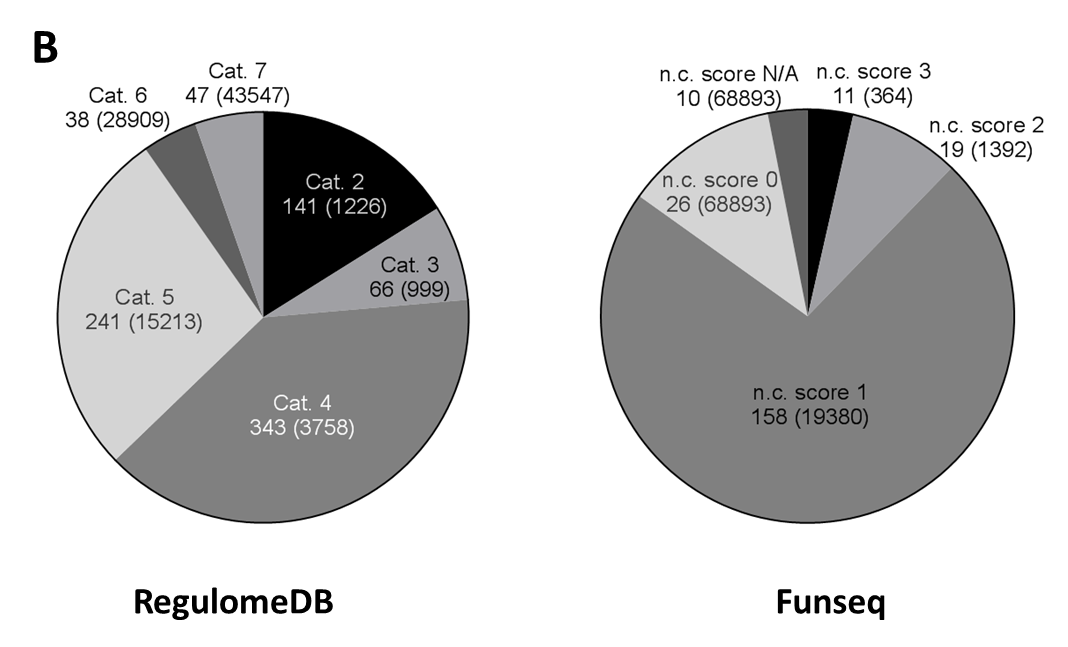


**Additional File 2 Figure.** Comparison of RegulomeDB and Funseq annotations against mutations annotated by OncoCis as being (A) associate with differential gene expression and (B) associated with the creation of a transcription factor binding site and also with a DHS. The number within each scoring category is shown with the total mutations within the corresponding category in brackets. Cat. = Category. n.c. score= non-coding score.

As expected, RegulomeDB and Funseq annotate the majority of these mutations with at least some regulatory features (i.e. Cat. 6 or less for RegulomeDB and non-coding score of 1 or greater for Funseq). In both cases, only a relatively small number of mutations annotated by OncoCis were annotated as non-functional by RegulomeDB or Funseq. This is likely due to the criteria of mutations having to be annotated within a DHS in this example. Significantly, for both differential gene expression and transcription factor binding creation, the majority of mutations fall within categories 5 & 6 for RegulomeDB and non-coding score 1 for Funseq.
