# Supplementary material for: OncoCis: annotation of cis-regulatory mutations in cancer
Source: Genome Biol. 2014 Oct 9;15(10):485. doi: 10.1186/s13059-014-0485-0 (PMC4224696; doi:10.1186/s13059-014-0485-0)
Supplement: Additional file 3: — Expression of THAP transcription factors across the breast cancer samples. [file 13059_2014_485_MOESM3_ESM.docx]

**Additional File 3.** Summary of expression values for all *THAP* factors across the 17 breast cancer samples. The sample (PD4107a) with the *CDK6* associated mutation is highlighted in red.

| **Gene** | **PD3851a** | **PD3890a** | **PD3904a** | **PD3905a** | **PD4005a** | **PD4006a** | **PD4085a** | **PD4086a** | **PD4088a** | **PD4103a** | **PD4107a** | **PD4109a** | **PD4115a** | **PD4116a** | **PD4192a** | **PD4198a** | **PD4248a** |
| --- | --- | --- | --- | --- | --- | --- | --- | --- | --- | --- | --- | --- | --- | --- | --- | --- | --- |
| *THAP1* | 270.2 | 523.1 | 321.8 | 318.7 | 320.7 | 434.4 | 578.6 | 354.8 | 663.8 | 816.3 | 450.8 | 567.7 | 397.0 | 811.1 | 210.6 | 482.9 | 360.9 |
| *THAP2* | 133.2 | 134.3 | 174.0 | 152.6 | 144.5 | 150.6 | 161.3 | 116.3 | 243.2 | 232.7 | 114.5 | 124.0 | 183.5 | 184.7 | 131.2 | 155.2 | 130.2 |
| *THAP3* | 141.8 | 145.5 | 119.6 | 144.0 | 166.8 | 118.9 | 140.4 | 150.8 | 194.9 | 154.5 | 142.8 | 204.4 | 133.5 | 113.3 | 137.6 | 182.9 | 127.8 |
| *THAP4* | 158.3 | 183.0 | 130.0 | 72.0 | 60.5 | 144.4 | 97.0 | 45.1 | 88.9 | 52.9 | 79.6 | 75.8 | 128.7 | 160.2 | 80.3 | 74.8 | 76.0 |
| *THAP5* | 166.5 | 147.5 | 149.6 | 153.5 | 212.8 | 171.9 | 159.4 | 157.2 | 172.1 | 236.0 | 192.9 | 166.4 | 176.3 | 169.5 | 304.4 | 241.1 | 195.6 |
| *THAP6* | 380.3 | 431.5 | 512.4 | 277.4 | 360.9 | 238.2 | 443.9 | 190.1 | 482.3 | 967.3 | 377.6 | 348.0 | 384.8 | 324.5 | 475.0 | 388.5 | 297.1 |
| *THAP7* | 210.7 | 511.3 | 390.2 | 364.3 | 448.4 | 406.0 | 556.2 | 292.5 | 300.3 | 432.8 | 370.1 | 443.6 | 355.7 | 440.4 | 455.4 | 281.4 | 585.7 |
| *THAP8* | 174.8 | 146.8 | 159.3 | 175.9 | 135.1 | 142.8 | 195.4 | 143.1 | 142.1 | 99.6 | 233.3 | 133.6 | 143.9 | 154.3 | 113.8 | 133.2 | 141.3 |
| *THAP9* | 50.6 | 65.2 | 125.6 | 63.3 | 39.8 | 70.6 | 67.0 | 67.2 | 71.9 | 74.8 | 65.3 | 77.3 | 54.3 | 54.3 | 47.4 | 37.7 | 34.0 |
| *THAP10* | 245.4 | 410.9 | 673.5 | 356.4 | 287.4 | 395.2 | 401.4 | 520.1 | 681.4 | 538.4 | 475.6 | 480.3 | 333.6 | 701.5 | 91.1 | 200.3 | 503.0 |
| *THAP11* | 2427.3 | 3276.1 | 1862.0 | 2233.4 | 1760.8 | 2190.8 | 1506.2 | 1642.0 | 1693.8 | 2299.5 | 2305.4 | 2502.4 | 2338.5 | 1452.0 | 1684.3 | 1352.5 | 985.5 |
