# Supplementary material for: OncoCis: annotation of cis-regulatory mutations in cancer
Source: Genome Biol. 2014 Oct 9;15(10):485. doi: 10.1186/s13059-014-0485-0 (PMC4224696; doi:10.1186/s13059-014-0485-0)
Supplement: Additional file 4: — Analysis of RNA-seq data to identify potential alternative TSS in CDK6. [file 13059_2014_485_MOESM4_ESM.docx]

**Additional File 4. RNA-seq analysis of *CDK6* to identify and potential alternative transcription start sites**

As FANTOM5 TSS annotation show a number of cell lines with evidence of transcription arising immediate downstream of the DHS containing the chr7:92,347,495 G>C substitution, we used RNA-seq data (European Genome-phenome Archive: EGAD00001001019) from the matching tumour of patient PD4107a to determine whether alternative *CDK6* transcripts are also present in this sample. To ensure that there are no alternative *CDK6* transcripts, the RNA-seq reads were aligned using three different methods (1) split read mapping to genome (tophat), (2) full read mapping to genome (bwa) and (3) split read mapping to transcriptome (RNA Architect). However, no evidence of alternative transcripts arising downstream of the mutation were found (Additional File 4 Figure 1). This suggests that the region does not act as a promoter in this sample.


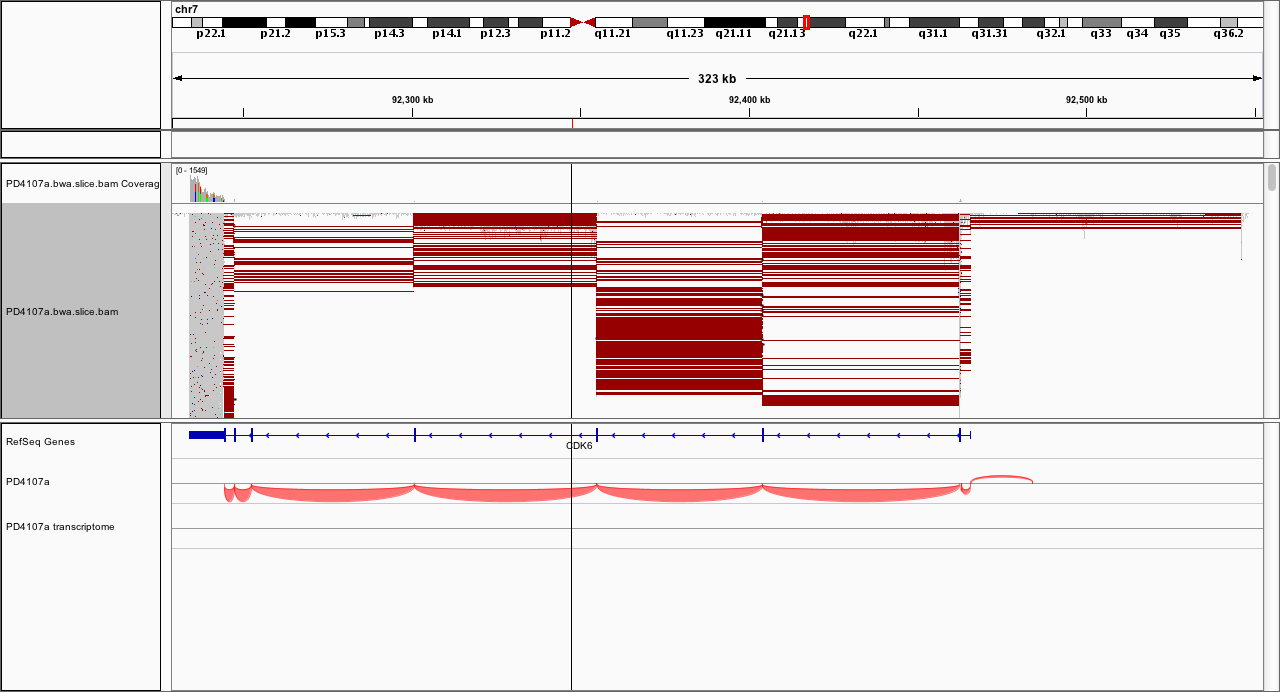


**Additional File 4 Figure 1.** RNA-seq reads mapping to CDK6 in sample PD4107a visualised using IGV. The location of the mutation is marked by the vertical black line.
