# Supplementary material for: OncoCis: annotation of cis-regulatory mutations in cancer
Source: Genome Biol. 2014 Oct 9;15(10):485. doi: 10.1186/s13059-014-0485-0 (PMC4224696; doi:10.1186/s13059-014-0485-0)
Supplement: Additional file 5: — Examples of other candidate mutations from the breast cancer dataset prioritised by OncoCis. [file 13059_2014_485_MOESM5_ESM.docx]

**Additional File 5. Illustration of other candidate mutations from the breast cancer dataset prioritised by OncoCis**

***IL6* chr7:22,617,382 C>T (PD4107a)**

Interleukin 6 (IL-6) is an interleukin that acts as inflammatory cytokine and is normally associated with haematopoiesis and lymphocyte activation [[1](#_ENREF_1)]. However, it is now also known that IL-6 plays an important role as a mediator of progression in many cancers [[2-5](#_ENREF_2)]. In breast cancer, it has been shown that elevated serum IL-6 correlates with poor disease outcome [[6](#_ENREF_6)]. It has also been shown that IL-6 levels in primary tumours are elevated relative to normal tissue [[7](#_ENREF_7), [8](#_ENREF_8)].

In this case, a mutation chr7:22,617,382 C>T is found within a putative enhancer upstream of *IL-6*. The association between the enhancer and *IL-6* is based on the FANTOM5 dataset [[9](#_ENREF_9)] (Figure 1A). The mutation potentially creates a CEBPA motif within the enhancer. CEBPA generally acts as a promoter of gene expression [[10](#_ENREF_10)]. It is known that CEBPA is an important regulator of *IL-6* at its promoter [[11](#_ENREF_11), [12](#_ENREF_12)]. The introduction of CEBPA binding at an *IL-6* enhancer can potentially increase *IL-6* expression which is consistent with the relatively high *IL-6* expression in PD4116a relative to other breast cancer samples (Figure 1B).

**Figure 1.** (A) Illustration of OncoCis annotation of a mutation associated with *IL6*. The FANTOM5 Enhancer-TSS association used to map the mutation to *IL6* is highlighted in yellow. (B) Expression of *IL6* among the 17 breast cancer samples. Dot in red is PD4107a with the potential cis-regulatory mutation.

***COX6C* chr8:100,811,550 T>A (PD4116a)**

Cytochrome c oxidase subunit 6C (COX6C) is a subunit of the mitochondrial respiratory enzyme cytochrome C oxidase. COX6C is one of the nuclear-encoded subunits thought to be important for the regulation and assembly of the cytochrome C oxidase complex [[13](#_ENREF_13)]. COX6C is commonly found as a fusion protein with HMGA2 in uterine leiomyoma [[14](#_ENREF_14)]. The most common fusion occurs with the fusion of the first three exons of *HMGA2* to exon 2 of *COX6C* [[15](#_ENREF_15)]. HMGA2 is a DNA binding protein that has been shown to be frequently up-regulated in cancers [[16-19](#_ENREF_16)]. Since the DNA binding domain of HMGA2 is within its first 3 exons, this suggests that *HMGA2* is more likely to be the oncogenic partner in the HMGA2-COX6C fusion protein. Nevertheless, *COX6C* has been shown to be up-regulated in prostate cancer cells [[20](#_ENREF_20)] and its dysregulation may play a role in altering energy metabolism which is a common feature of cancer cells [[21](#_ENREF_21)]. In this case, the chr8:100,811,550 T>A mutation falls within a possible enhancer for *COX6C* (Figure 2A). The mutation potentially abolishes NFKB1 binding at this enhancer. NFKB1 can act as both an activator and a repressor [[22](#_ENREF_22)] and thus, it is possible that the loss of NFKB1 binding can lead to increase *COX6C* expression which is significantly higher in the sample with the mutation compared with all others (Figure 2B).

**Figure 2.** (A) Illustration of OncoCis annotation of mutation associated with *COX6C*. (B) Expression of *COX6C* among the 17 breast cancer samples. Dot in red is PD4116a with the potential cis-regulatory mutation.

***HIC1* chr17:2,080,270 G>A (PD4005a)**

Hypermethylated in cancer 1 (*HIC1*) is a gene that is ubiquitously expressed at high levels across many tissue types but its expression is often absent or decreased in solid tumours [[23](#_ENREF_23)]. The gene functions as a transcriptional repressor and it has been shown to function as a tumour suppressor [[24-26](#_ENREF_24)].

In this case, the mutation chr17:2,080,270 G>A falls within the intron of *SMG6* but has mapped to *HIC1* as the closest gene by GREAT using OncoCis (Figure 3A). The mutation creates a NFIC motif. The mutation is linked to an increase in gene expression of *HIC1* (Figure 3B) which implies that the mutation may be a passenger mutation as *HCI1* is a tumour suppressor. This example again demonstrates the ability of OncoCis to link mutations to potential gene dysregulation, however it highlights the need for further verification of the relevance of the mutation in the context of driving cancer.

**Figure 3.** (A) Illustration of OncoCis annotation of mutation associated with *HIC1*. (B) Expression of *HIC1* among the 17 breast cancer samples. Dot in red is PD4005a with the potential cis-regulatory mutation.

**References**

1. Scheller J, Chalaris A, Schmidt-Arras D, Rose-John S: **The pro- and anti-inflammatory properties of the cytokine interleukin-6.** *Biochimica et biophysica acta* 2011, **1813:**878-888.

2. Chen MF, Chen PT, Lu MS, Lin PY, Chen WC, Lee KD: **IL-6 expression predicts treatment response and outcome in squamous cell carcinoma of the esophagus.** *Molecular cancer* 2013, **12:**26.

3. Nagasaki T, Hara M, Nakanishi H, Takahashi H, Sato M, Takeyama H: **Interleukin-6 released by colon cancer-associated fibroblasts is critical for tumour angiogenesis: anti-interleukin-6 receptor antibody suppressed angiogenesis and inhibited tumour-stroma interaction.** *British journal of cancer* 2014, **110:**469-478.

4. Dethlefsen C, Hojfeldt G, Hojman P: **The role of intratumoral and systemic IL-6 in breast cancer.** *Breast cancer research and treatment* 2013, **138:**657-664.

5. Belluco C, Nitti D, Frantz M, Toppan P, Basso D, Plebani M, Lise M, Jessup JM: **Interleukin-6 blood level is associated with circulating carcinoembryonic antigen and prognosis in patients with colorectal cancer.** *Annals of surgical oncology* 2000, **7:**133-138.

6. Salgado R, Junius S, Benoy I, Van Dam P, Vermeulen P, Van Marck E, Huget P, Dirix LY: **Circulating interleukin-6 predicts survival in patients with metastatic breast cancer.** *International journal of cancer Journal international du cancer* 2003, **103:**642-646.

7. Culig Z, Steiner H, Bartsch G, Hobisch A: **Interleukin-6 regulation of prostate cancer cell growth.** *Journal of cellular biochemistry* 2005, **95:**497-505.

8. Garcia-Tunon I, Ricote M, Ruiz A, Fraile B, Paniagua R, Royuela M: **IL-6, its receptors and its relationship with bcl-2 and bax proteins in infiltrating and in situ human breast carcinoma.** *Histopathology* 2005, **47:**82-89.

9. Andersson R, Gebhard C, Miguel-Escalada I, Hoof I, Bornholdt J, Boyd M, Chen Y, Zhao X, Schmidl C, Suzuki T, et al: **An atlas of active enhancers across human cell types and tissues.** *Nature* 2014, **507:**455-461.

10. Miller M, Shuman JD, Sebastian T, Dauter Z, Johnson PF: **Structural basis for DNA recognition by the basic region leucine zipper transcription factor CCAAT/enhancer-binding protein alpha.** *The Journal of biological chemistry* 2003, **278:**15178-15184.

11. Zhang P, Iwama A, Datta MW, Darlington GJ, Link DC, Tenen DG: **Upregulation of interleukin 6 and granulocyte colony-stimulating factor receptors by transcription factor CCAAT enhancer binding protein alpha (C/EBP alpha) is critical for granulopoiesis.** *The Journal of experimental medicine* 1998, **188:**1173-1184.

12. Faggioli L, Costanzo C, Donadelli M, Palmieri M: **Activation of the Interleukin-6 promoter by a dominant negative mutant of c-Jun.** *Biochimica et biophysica acta* 2004, **1692:**17-24.

13. Otsuka M, Mizuno Y, Yoshida M, Kagawa Y, Ohta S: **Nucleotide sequence of cDNA encoding human cytochrome c oxidase subunit VIc.** *Nucleic acids research* 1988, **16:**10916.

14. Kurose K, Mine N, Doi D, Ota Y, Yoneyama K, Konishi H, Araki T, Emi M: **Novel gene fusion of COX6C at 8q22-23 to HMGIC at 12q15 in a uterine leiomyoma.** *Genes, chromosomes & cancer* 2000, **27:**303-307.

15. Mine N, Kurose K, Nagai H, Doi D, Ota Y, Yoneyama K, Konishi H, Araki T, Emi M: **Gene fusion involving HMGIC is a frequent aberration in uterine leiomyomas.** *Journal of human genetics* 2001, **46:**408-412.

16. Meyer B, Loeschke S, Schultze A, Weigel T, Sandkamp M, Goldmann T, Vollmer E, Bullerdiek J: **HMGA2 overexpression in non-small cell lung cancer.** *Molecular carcinogenesis* 2007, **46:**503-511.

17. Di Cello F, Hillion J, Hristov A, Wood LJ, Mukherjee M, Schuldenfrei A, Kowalski J, Bhattacharya R, Ashfaq R, Resar LM: **HMGA2 participates in transformation in human lung cancer.** *Molecular cancer research : MCR* 2008, **6:**743-750.

18. Ding X, Wang Y, Ma X, Guo H, Yan X, Chi Q, Li J, Hou Y, Wang C: **Expression of HMGA2 in bladder cancer and its association with epithelial-to-mesenchymal transition.** *Cell proliferation* 2014, **47:**146-151.

19. Bartuma H, Panagopoulos I, Collin A, Trombetta D, Domanski HA, Mandahl N, Mertens F: **Expression levels of HMGA2 in adipocytic tumors correlate with morphologic and cytogenetic subgroups.** *Molecular cancer* 2009, **8:**36.

20. Wang FL, Wang Y, Wong WK, Liu Y, Addivinola FJ, Liang P, Chen LB, Kantoff PW, Pardee AB: **Two differentially expressed genes in normal human prostate tissue and in carcinoma.** *Cancer research* 1996, **56:**3634-3637.

21. Moreno-Sanchez R, Rodriguez-Enriquez S, Marin-Hernandez A, Saavedra E: **Energy metabolism in tumor cells.** *The FEBS journal* 2007, **274:**1393-1418.

22. Tong X, Yin L, Washington R, Rosenberg DW, Giardina C: **The p50-p50 NF-kappaB complex as a stimulus-specific repressor of gene activation.** *Molecular and cellular biochemistry* 2004, **265:**171-183.

23. Fleuriel C, Touka M, Boulay G, Guerardel C, Rood BR, Leprince D: **HIC1 (Hypermethylated in Cancer 1) epigenetic silencing in tumors.** *The international journal of biochemistry & cell biology* 2009, **41:**26-33.

24. Zhang W, Zeng X, Briggs KJ, Beaty R, Simons B, Chiu Yen RW, Tyler MA, Tsai HC, Ye Y, Gesell GS, et al: **A potential tumor suppressor role for Hic1 in breast cancer through transcriptional repression of ephrin-A1.** *Oncogene* 2010, **29:**2467-2476.

25. Pinte S, Stankovic-Valentin N, Deltour S, Rood BR, Guerardel C, Leprince D: **The tumor suppressor gene HIC1 (hypermethylated in cancer 1) is a sequence-specific transcriptional repressor: definition of its consensus binding sequence and analysis of its DNA binding and repressive properties.** *The Journal of biological chemistry* 2004, **279:**38313-38324.

26. Boulay G, Malaquin N, Loison I, Foveau B, Van Rechem C, Rood BR, Pourtier A, Leprince D: **Loss of Hypermethylated in Cancer 1 (HIC1) in breast cancer cells contributes to stress-induced migration and invasion through beta-2 adrenergic receptor (ADRB2) misregulation.** *The Journal of biological chemistry* 2012, **287:**5379-5389.
