# Supplementary material for: OncoCis: annotation of cis-regulatory mutations in cancer
Source: Genome Biol. 2014 Oct 9;15(10):485. doi: 10.1186/s13059-014-0485-0 (PMC4224696; doi:10.1186/s13059-014-0485-0)
Supplement: Additional file 6: — Accession of datasets implement in OncoCis. [file 13059_2014_485_MOESM6_ESM.docx]

**Additional File 6.** Summary of all datasets implemented in the OncoCis tool. The GEO accession is shown for all datasets. All datasets are from the ENCODE project except those marked with an asterisk (*) which are from the Human Epigenome Atlas.

| **Cell/tissue type** | **Cell line name** | **Description** | **DHS** | **H3K4me1** | **H3K4me3** | **H3K27ac** |
| --- | --- | --- | --- | --- | --- | --- |
| Lung | A549 | Alveolar basal epithelial adenocarcinoma | GSM736580 | GSM1003453 | GSM1003578 | GSM1003578 |
| Prostate | LNCaP | Prostate epithelial adenocarcinoma | GSM736603 | GSM353634 | GSM945240 | GSM686938 |
| Liver | HepG2 | Hepatocellular epithelial carcinoma | GSM736639 | GSM798321 | GSM733737 | GSM733743 |
| Blood | K562 | Chronic myelogenous leukemia | GSM736629 | GSM733692 | GSM733680 | GSM733656 |
| Blood | CD34 | Normal CD34+ mobilised hematopoeitic stem/progenitor cells | GSM530658* | GSM706845* | GSM621437* | GSM772885* |
| Breast | HMEC | Normal human mammary epithelial cells | GSM753973* | GSM613876* | GSM613874* | GSM733660 |
| Melanocytes | Melano | Normal foreskin melanocytes | GSM774244* | GSM958152* | GSM958151* | GSM958157* |
| Cervical | HeLa | Cervival epithelial adenocarcinoma | GSM736564 | GSM798322 | GSM733682 | GSM733684 |
| Colon | HCT116 | Colon epithelial carcinoma | GSM736493 | GSM945858 | GSM945304 | GSM945853 |
| Pancreas | PANC-1 | Pacreatic epithelioid carcinoma | GSM736519 | GSM818827 | GSM945856 | GSM818826 |
| Astrocytes | NHA | Normal human astrocytes | GSM736544 | GSM733710 | GSM733747 | GSM733763 |
| Osteoblast | Osteo | Normal human osteoblasts | GSM816654 | GSM733704 | GSM1003466 | GSM733739 |
| Mesenchymal stem cell | MSC | Human mesenchymal stem cell, differentiated from H1 ES cells | GSM906380* | GSM753437* | GSM767348* | GSM767341* |
| Neural progenitor cell | NPC | Human neural progenitor cells, differentiated from H1 ES cells | GSM878615* | GSM818039* | GSM767350* | GSM753429* |
| Embryonic stem cell | ESC | Human embryonic stem cells, undifferentiated | GSM736582 | GSM733782 | GSM733657 | GSM733718 |
