# Supplementary material for: OncoCis: annotation of cis-regulatory mutations in cancer
Source: Genome Biol. 2014 Oct 9;15(10):485. doi: 10.1186/s13059-014-0485-0 (PMC4224696; doi:10.1186/s13059-014-0485-0)
Supplement: Additional file 7: — Illustrating the distribution of histone profiles adjacent to DHSs. [file 13059_2014_485_MOESM7_ESM.docx]

**Additional File 7** Average profile of histone marks (H3K4me1, H3K4me3 and H3K27ac) relative to average DHS peak profiles.
