# Supplementary material for: OncoCis: annotation of cis-regulatory mutations in cancer
Source: Genome Biol. 2014 Oct 9;15(10):485. doi: 10.1186/s13059-014-0485-0 (PMC4224696; doi:10.1186/s13059-014-0485-0)
Supplement: Additional file 8: — Analysis of the effect of number of well conserved positions in motifs against its frequency of being found to be created/removed. [file 13059_2014_485_MOESM8_ESM.docx]

**Additional File 8. The number of well conserved residues in a motif does not impact on the likelihood of OncoCIS annotating a mutation as either creating or deleting that motif.**

To ensure that the use of a filter based on the number of well conserved bases within a motif does not significantly affect the probability with which the motif is found, the following analysis was performed. We evaluated the frequency with which a motif is identified as being created/removed in relation to the number of well conserved positions in that motif in relation to all mutations in the test breast cancer dataset.

The number of motifs that were created or removed did not correlate with the number of conserved bases, but followed a general Gaussian pattern. Motifs with three conserved bases were identified most frequently as being created or removed (Additional file 8 Figure 1A). This mirrors the distribution of motifs matching the wild-type or mutant sequence based purely on log-likelihood ratio score (Additional file 8 Figure 1B). This suggests that the frequency with which a motif is created or removed is most dependent on the characteristics of the motif and rather than the number of well conserved bases. A scatterplot of the frequency of a motif being created/removed versus total number of matches confirms the strong correlation (r^2^ = 0.8411) (Additional file 8 Figure 1C).

**Additional file 8 Figure 1.** Influence of using well conserved bases (> 0.8) to identify motifs being created or removed across the breast cancer mutation dataset. (A) The distribution of the number of times motifs are created or removed categorised by the number of well conserved bases within each motif. (B) The distribution of the number of times motifs were matched to the wild-type or mutant DNA sequence based on log-likelihood ratio score alone categorised by the number of well conserved bases within each motif. (C) Correlation of the number of times each motif is created or removed vs. simply matching the wild-type or the mutant sequence.
